# Luminescent Zn_2_GeO_4_:Mn^2+^ Nanoparticles with High Quantum Yield for Salivary Protein Detection

**DOI:** 10.1021/acsanm.5c02725

**Published:** 2025-08-10

**Authors:** Yuchen Zhang, Yuanbing Mao

**Affiliations:** † Department of Chemistry, 2455 Illinois Institute of Technology, Chicago, Illinois 60616, United States

**Keywords:** zinc germanate, nanoparticles, photoluminescence, biosensing, immunoassay

## Abstract

Zinc germanate doped with Mn^2+^ (Zn_2_GeO_4_:Mn^2+^) is known to be a green luminescence phosphor with many applications in biosensing and bioimaging. This study presents a simple method for creating small size Zn_2_GeO_4_:Mn^2+^ nanoparticles using a combination of the coprecipitation–molten salt synthesis method. These nanoparticles exhibit bright green luminescence under UV excitation. After surface functionalization, these nanoparticles were then used to develop a fluorescence resonance energy transfer (FRET)-based immunoassay. This immunoassay shows a detection range of 5–20 ng/mL of C-reactive protein (CRP), which suggests its potential for simple solution CRP detection and broader applications in protein biosensing.

## Graphical abstract

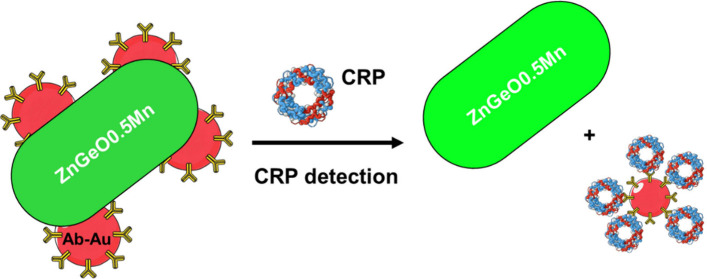

## Introduction

The conversion of excitation energy to emitted photons of luminescent materials is usually not 100% efficient due to reasons causing losses through alternative deactivation pathways. Common reasons include nonradiative transitions, self-absorption, impurities and defects, etc.^1^ Researchers continuously work on improving luminescent materials for achieving higher efficiency and better performance in lighting, displays, sensors, and other fields.^1^ Some key strategies to enhance luminescence efficiency include new material design, purity and quality control, additional doping, etc.^2^

With different host and doping materials, Zn_2_GeO_4_:Mn^2+^ (ZGOM) photoluminescent (PL) nanoparticles (NPs) have been explored as promising candidates for biosensing applications. This particular material exhibits green luminescence under UV excitation.^3^ Researchers have employed different techniques to control their size, shape, and aspect ratio. For example, rod-like Zn_2_GeO_4_ (ZGO) NPs have been synthesized using various methods. Hydrothermal/solvothermal synthesis have been used to make rod-like ZGOM NPs by controlling reaction conditions.^3^ Microwave-assisted hydrothermal synthesis has also been used to generate ZGOM NPs with reduced reaction time.^4^ Although controlled particle size and shape of ZGOM NPs have been successfully synthesized, postannealing is still required to achieve efficient PL.^5^ With regular postannealing, NPs may agglomerate, grow in particle size, and reduce dispersibility in water for potential bioapplications.^6^

Compared to high temperature solid-state synthesis, hydrothermal synthesis, and annealing methods, molten salt synthesis (MSS) is a desirable method for inorganic phosphors.^7^ In the molten state of inorganic salts, ions are free to move and can act as reaction media for materials synthesis. They serve as ionic solvents to enhance precursors’ dissociation into constituent anions and cations and form desirable products. These high temperature solvents have the advantage of high heat capacity, wide range of operation temperature, good fluidity, and high chemical and thermal stability as desirable high-temperature reaction media.^8^ Moreover, ions inside molten salt media can act as surface protection agents to prevent particle agglomeration during high temperatures process.^8^ NPs synthesized by the MSS method generally do not need postsynthesis annealing while possessing desirable properties.

Meanwhile, human biofluids play essential roles in maintaining health and providing valuable diagnostic information, such as blood including plasma and serum, saliva, urine, and sweat.^9^ Among them, serum gives one of the widest variety of biomarkers for different diagnostics with accurate information. However, sample preparation and tests of serum require specialists with authorization.^10^ In recent years, saliva has received increasing consideration with many advantages over serum. For example, saliva can be collected by noninvasive methods, which generalists could collect samples by themselves without depending on specialists while reducing infection risk from needles or bloodborne pathogens.^11^ Many researchers have been working on salivary diagnostic such as HIV-1 and -2; viral hepatitis A, B, and C; and SARS-CoV-2.^12^ Dentists also pay attention to salivary diagnostics for potential disease prediction and detection including periodontitis.^13^

C-reactive protein (CRP) is a widely used biomarker for assessing systemic inflammation. It is routinely measured in serum or plasma samples and plays a crucial role in clinical medicine and research. CRP in saliva has some correlation to serum CRP level and can be tested for preclinical evaluation.^14^ For example, in blood samples, less than 3 mg/L of CRP is considered as the normal level and over 10 mg/mL as moderate elevation with systemic inflammation.^14^ In saliva samples, ∼3 ng/mL of CRP is considered as normal and over 10 ng/mL as moderate elevation.^14^ Several CRP detection methods already exist such as high-sensitivity CRP (hs-CRP) and enzyme-linked immunosorbent assay (ELISA) tests. They require serum samples or take time and, therefore, are not suitable for simple preclinical evaluation. Hence, new methods for salivary CRP detection are sought for fast CRP testing.

Herein, we synthesized ZGOM NPs by novel combined coprecipitation–MSS processes with controlled particle size and bright photoluminescence. We explored their potential application for CRP sensing after functionalizing them with antibody-coated gold NPs based on the fluorescence resonance energy transfer (FRET) effect (Scheme 1). We showed a CRP detection range of 5–20 ng/mL and qualitatively 2.5 ng/mL of CRP concentration in a simple solution environment without further separation. This FRET-based immunoassay is a desirable candidate for rapid salivary CRP detection.

**Scheme 1:**
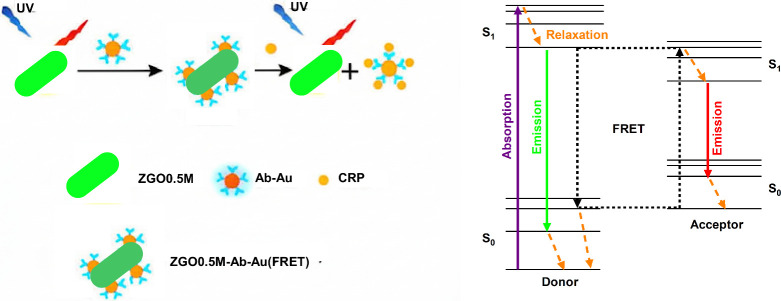
Immunoassay of CRP Detection Based on the FRET Effect

## 2 Results and Discussions

### 2.1 Optimizing Synthesis Condition for ZGOM NPs

We first optimized the synthesis conditions (SI S1) to maximize the luminescence performance of our as-synthesized ZGOM NPs. Specifically, we first optimized Mn^2+^ doping concentrations and obtained pure ZGO:x%Mn^2+^ NPs with 0.5% Mn^2+^ doping level (ZGO0.5M) showing the highest PL emission at 535 nm from Mn^2+^ luminescence with excitation at 254 nm (Figure S1). We also studied the effects of MSS temperature and duration and achieved pure ZGO0.5M NPs with the highest PL intensity for MSS at 900 °C for 2 h (Figures S2 and S3). Further, the ZGO0.5M NPs synthesized from the precursor coprecipitated at pH 10 (Figure S4) showed the best PL performance with pure XRD patterns. Different XRD patterns may be due to the formation of precursors coprecipitated at various pH values, i.e., GeO_2_ at pH < 9 and H_3_GeO_4_^–^ at pH > 9.^15^ We also studied the effect of the molar ratio of precursor to NaCl and KCl mixture and obtained the highest PL from the ZGO0.5M NPs synthesized with the ratio of precursors:NaCl:KCl = 1:10:10 (Figure S5). A Raman spectrum also confirmed the formation of the desired ZGO structure (Figure S6).

Zn_2_GeO_4_ normally crystallizes in the rhombohedral crystal system with space group *R̅*3. Its crystal structure consists of alternating [GeO_4_] and [ZnO_4_] tetrahedra arranged in a pattern parallel to the *c*-axis. Pure ZGO0.5M NPs (Figure 1a) were successfully synthesized under the optimized conditions via the combined coprecipitation and MSS process, i.e., ZGO0.5M NPs with a Mn^2+^ doping level of 0.5% by the MSS process upon heating the precursor coprecipitated at pH 10 at 900 °C for 2h with a molar ratio of the precursor to mixed NaCl/KCl at 1:10:10. Rietveld refinement results of ZGO0.5M NPs (Figure 1a) indicated that Mn^2+^ doping does not distort the basic ZGO structure. According to the ionic size and charge, Mn^2+^ doping ions (ionic radius = 66 pm, CN = 4) are expected to substitute Zn^2+^ ions (ionic radius = 60 pm, CN = 4) in the [ZnO_4_] site while the [GeO_4_] site (ionic radius of Ge = 53 pm, CN = 4) is too small for Mn^2+^ substitution. The crystal structure of the ZGO0.5M NPs based on the Rietveld refinement results (Figure 1b) demonstrates that both [ZnO_4_] and [GeO_4_] sites are present in the structure. SEM image (Figure 1c) confirms that the ZGO0.5M NPs have short nanorod shape with an average diameter of ∼100 nm and a length ∼300 nm. According to Hartman and Perdok’s theory,^16^ crystal growth tends to occur along directions that involve the formation of strong bond chains. As a result, ZGO crystals are more likely to grow preferentially in the *c*-axis direction, leading to the formation of rod-like crystals by alignment of the tetrahedral chains with the interaction of the used chloride molten salt.^22^ TEM and SAED results (Figure S7) also confirmed the morphology and crystal lattice of ZGO with the (113) plane present in the HRTEM image. EDS data (Figure S8) confirmed the existence and even distribution of the composing elements in our ZGOM NPs.

**Figure 1:**
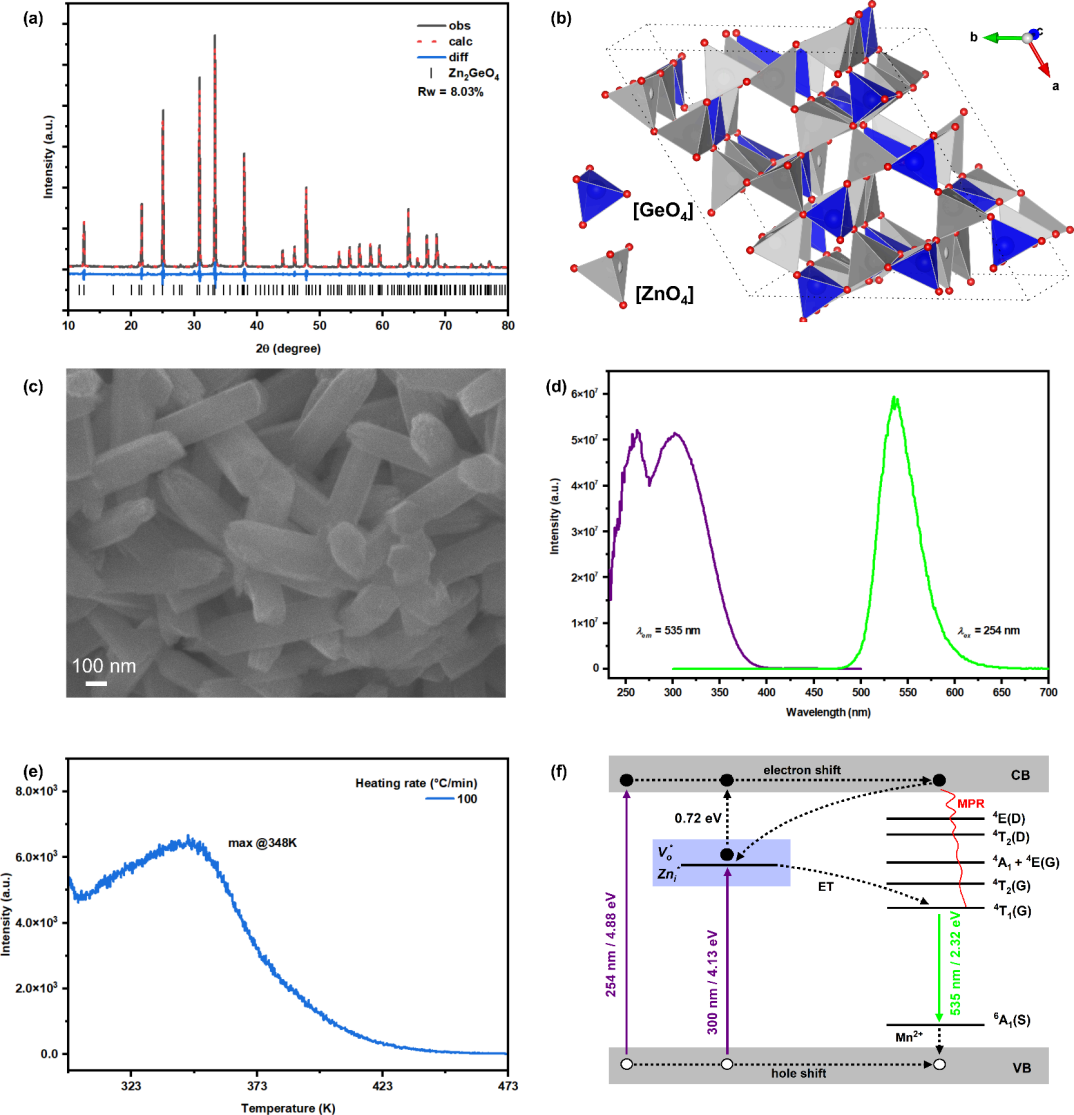
ZGO0.5M NPs synthesized under the optimized conditions: (a) XRD pattern and Rietveld refinement along with JCPDS No. 110687. (b) Crystal structure of ZGO built from the Rietveld refinement results. (c) SEM image. (d) PL and PLE spectra taken at room temperature with λ_ex_ = 254 nm and λ_em_ = 535 nm. (e) TL spectrum taken after being charged at 254 nm for 5 min. (f) Proposed energy diagram.

A typical PLE spectrum of the optimally synthesized ZGO0.5M NPs (Figure 1d) indicates two excitation bands: 254 nm peak is the band-to-band energy transfer (ET) from VB to CB of the ZGO host and the 300 nm peak come from the ET of VB of the ZGO host to defect energy levels such as *V*_o_^*^ or *Zn*_i_^*^.^4^ The relevant PL spectrum confirms Mn^2+^ emission from ^4^T_1_ to ^6^A_1_. To understand the energy structure, the TL spectrum of the ZGO0.5M NPs (Figure 1e) was taken showing shallow traps around 358 K. The corresponding trap depth was calculated to be 0.72 eV (Figure S11), which is consistent with the observed persistent luminescence (PersL) emission from these NPs (Figure S9).

Based on the PL, PLE, TL and PersL data discussed above, we proposed an energy diagram for our ZGO0.5M NPs (Figure 1f). First, electrons are excited to the CB of the ZGO0.5M NPs with UV light. The resulting excited electrons would either go back to the excited state of Mn^2+^ activator to generate PL emission or be trapped inside of the traps. The latter gives out PersL as the trapping electrons move back to the CB after the charging UV light was turned off.

QY measurements on our ZGO0.5M NPs upon excitation at 258 and 300 nm (Figures S10 and S14 and Table S1) gave calculated QY of 34.3%, which indicates highly efficient ET from the CB of ZGO host to Mn^2+^ activator, alone with strong back ET from shallow traps.^17^

To confirm the advantage of our coprecipitation–MSS method for synthesizing ZGOM NPs, we also employed multiple published synthesis methods with minor modifications to synthesize ZGO0.5M samples (Table S1 and SI S2), including direct MSS,^6^ hydrothermal-MSS, hydrothermal-anneal,^18^ and hydrothermal methods.^19^ Pure ZGO0.5M samples were synthesized by these methods based on XRD data (Figure S12) except the minor ZnO impurity present in the sample synthesized by the direct MSS method. PL results confirmed that the ZGO0.5M NPs synthesized by our coprecipitation–MSS method possess the highest PL intensity (Figure S13) and QY value (Figure S14).

Synthesis methods of Zn_2_GeO_4_:Mn phosphors influence their luminescent properties. The solid-state reaction method, which involves high-temperature calcination of raw materials like ZnO, GeO_2_, and MnO_2_, produces phosphors with large particles and broad size distributions exhibiting green emission bands.^17^ The coprecipitation method, which precipitates precursor materials from solution followed by calcination, yields phosphors with relative uniform particle sizes and improved PL properties.^20^ Hydrothermal synthesis utilizing self-generated high pressure by aqueous reaction solution in closed vessels can produce Zn_2_GeO_4_:Mn^2+^ nanorods with bright green luminescence under ultraviolet irradiation with a QY of 5.3%.^3^ However, it could give uneven distribution of activators in the formed NPs, specifically Cr dopants on the surface of the NPs.^21^

From the as-compared results above and previously published reports, it should be noted that high-temperature synthesis or annealing process was required to improve the PL performance of ZGOM phosphors.^3^ Molten salt media could achieve such high temperature and is suitable for this process. Meanwhile, molten salts as reaction media can enhance the reaction speed and get homogeneous mixtures, similar to the general solution synthesis.^6^ A high ionic environment of molten salt could also prevent agglomeration, which eliminates or reduces the formation of large particles. With all of these advantages of our coprecipitation–MSS method reported here, we achieved the best ZGOM NPs with the highest QY value compared to other methods.

### 2.2 Conjugated ZGO0.5M NPs for Salivary CRP Detection

For salivary CRP detection, we developed a CRP immunoassay as presented in Scheme 1. First [ZGO0.5M-Ab-Q] conjugation was formed by a mixture of the ZGO0.5M NPs and Ab-Q pair, where ZGO0.5M represents the coated ZGO0.5M-NH_2_ NPs, Ab the CRP antibody used to capture CRP protein, and Q the gold NPs as the quencher to quench the PL emission from the coated ZGO0.5M-NH_2_ NPs (ZGO0.5M).

The successful −NH_2_ functionalization of our ZGO0.5M NPs was confirmed by FTIR data (Figure S15). After the surface modification of the ZGO0.5M NPs with APTES, stretching and bending modes of the N–H bond at ∼3400 and 1600 cm^–1^ were presented in the IR spectrum. These peaks indicated that APTES was successfully coated outside of the ZGO0.5M NPs to generate the −NH_2_ end group. From the DLS data of the Au NPs (Figure S16­(a)), the hydrodynamic diameter of the Au NPs is around 7–9 nm, which is consistent with the localized surface plasmon resonance (LSPR) of Au NPs based on the absorption spectrum (Figure 2a). Surface-coated ZGOM NPs were stable in cold solution for more than a year (Figure S17).

**Figure 2:**
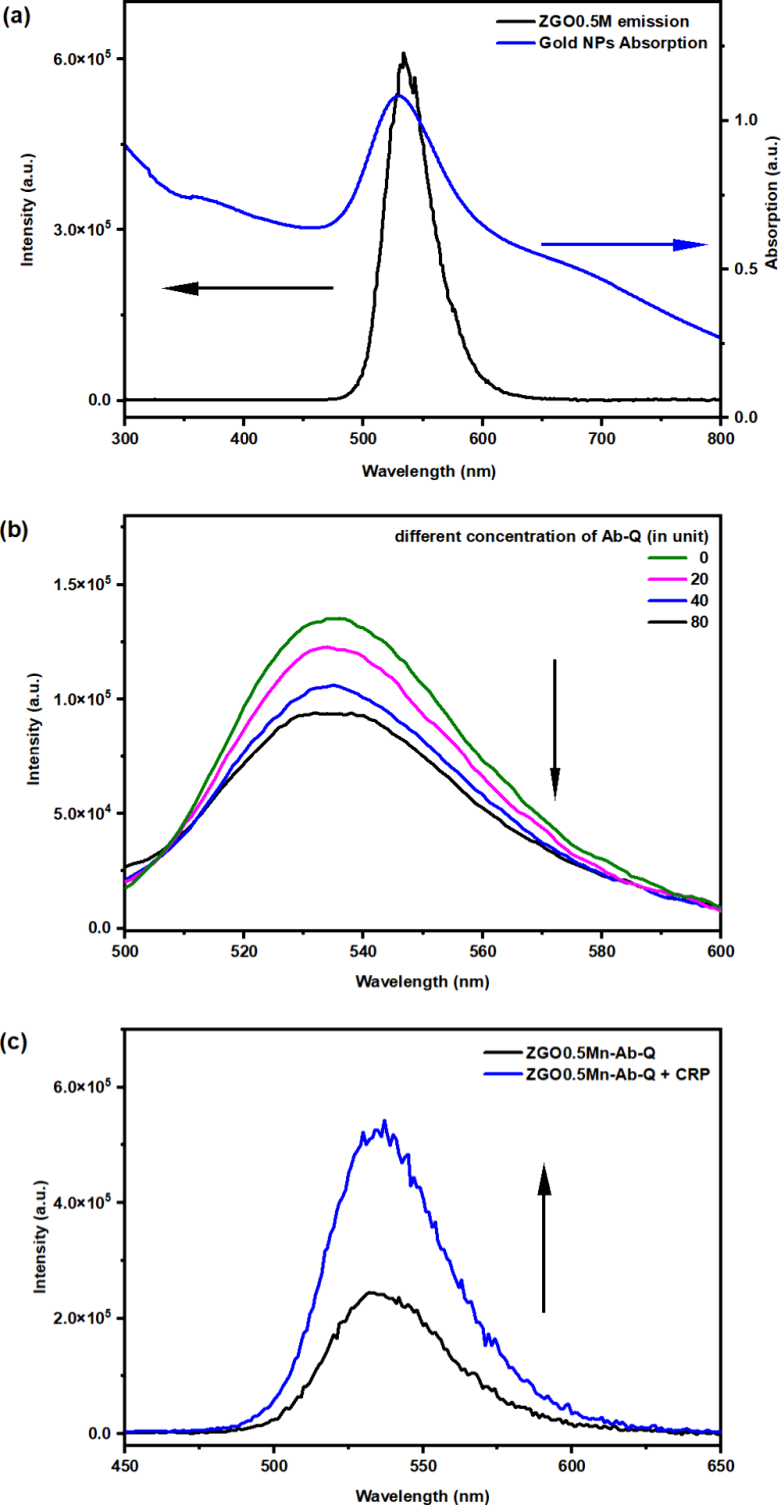
(a) PL emission spectrum of the ZGO0.5M NPs and absorption spectrum of our as-synthesized Au NPs. (b) PL emission intensity of the ZGO0.5M NPs decreased upon conjugation with Ab-Q. (c) PL emission recovery of the ZGO0.5M NPs upon adding CRP to the formed [ZGO0.5M-Ab-Q] conjugate solution.

The stability of the CRP antibody-coated gold NPs dispersed in various solutions were tested (Figure S18 and Table S2) within 10% NaCl solution.^22^ Upon coating with the CRP antibody, the surface of the Au NPs would be modified by the CRP antibody with electrostatic forces, which could prevent the Au NPs from agglomerating upon introducing high ionic solutions (such as 10% NaCl). These results confirmed the strong interaction between Au NPs and the CRP antibody.

The PL emission spectrum of the ZGO0.5M NPs and the absorption spectrum of our as-synthesized Au NPs are shown in Figure 2a. The maximum emission from our ZGO0.5M NPs was around 535 nm and highly overlapped with the absorption of the Au NPs, which indicated the optimized conditions for desirable FRET performance. As the [ZGO0.5M-Ab-Q] conjugation was formed due to electrostatic interactions among the components, the FRET effect was enhanced, while the PL intensity of the ZGO0.5M NPs was reduced. Due to the shortened distance between the ZGO0.5M NPs and Au NPs upon conjugating Ab-Q to them, the PL intensity of the ZGO0.5M NPs was reduced. Figure 2b shows the decreasing PL intensity due to the introduction of the Ab-Q pair.

When adding CRP to the as-formed [ZGO0.5M-Ab-Q] conjugate, the CRP molecules have stronger interaction with the Ab due to their antigen–antibody reaction, and the ZGO0.5M NPs got released from the Ab-Q pair. As the distance between the ZGO0.5M NPs and the Ab-Q pair increased, the PL emission of the ZGO0.5M NPs was recovered (Figure 2c). If there was no CRP protein added into the formed [ZGO0.5M-Ab-Q] conjugate solution, the [ZGO0.5M-Ab-Q] conjugate would remain intact, and the ZGO0.5M NPs would still be quenched. In other words, their PL emissions will not be recovered. We also confirmed the decrease of PL intensity based on the FRET effect by adding uncoated gold NPs (Figure S19). Without surface antibody modification, PL intensity would not change much by additional gold NPs due to the relatively weak interaction with the ZGOM NPs. After surface coating, surface-charged ZGOM and Ab-Q (Figure S16b, c) could have stronger interactions and form the ZGOM-Ab-Q conjugation (Figure S16d).

We took PL spectra of the as-formed [ZGO0.5M-Ab-Q] conjugate FRET immunoassay in the presence of CRP in the concentration range 5–20 ng/mL in the PBS (pH = 7.4) buffer solution (Figure 3a). As the result of introducing the targeted CRP, the PL emission of the formed [ZGO0.5M-Ab-Q] conjugate presented a clear recovery due to the desorption of the Ab-Q pair from the ZGO0.5M NPs. Based on the plotted standard curve between the relative intensity Δ*I*/*I*_0_ of the formed [ZGO0.5M-Ab-Q] conjugate and the concentration of added CRP (Figure 3b), a linear relationship within the concentration range of CRP from 5 to 20 ng/mL was obtained. This indicates that our [ZGO0.5M-Ab-Q] conjugate FRET immunoassay is suitable for salivary CRP detection in the concentration range with moderate elevation.^14^

**Figure 3:**
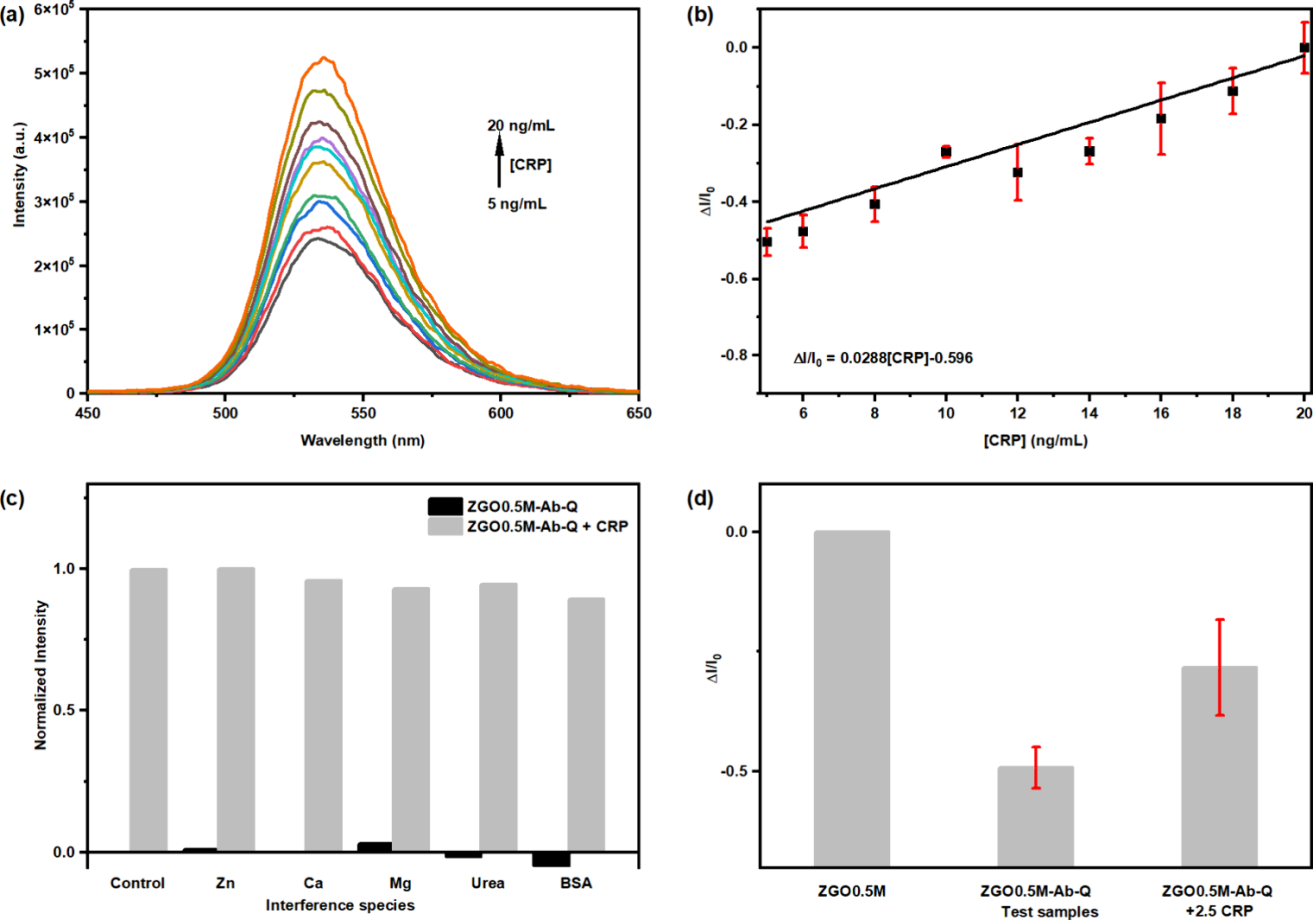
[ZGO0.5M-Ab-Q]-CRP immunoassay developed from our ZGO:0.5%Mn^2+^ NPs as a detection probe for salivary CRP by FRET effect. (a) PL recovery function with increasing CRP concentration. (b) Normalized standard curve of relative intensity difference Δ*I*/*I*_0_ vs CRP concentration over the range of 5 to 20 ng/mL. (c) Interference test of the developed [ZGO0.5M-Ab-Q] probe with different species as a generally presented saliva system. Black bar: PL intensity of the [ZGO0.5M-Ab-Q] probe mixed with different species including Zn^2+^, Ca^2+^, Mg^2+^, urea, and BSA. Red bar: PL intensity of the [ZGO0.5M-Ab-Q] probe mixture with these species followed with the addition of the CRP protein. The PL intensity was normalized based on the control sample which is from PBS/water solution. (d) Qualitive test of CRP protein at concentration around 2.5 ng/mL with diluted [ZGO0.5M-Ab-Q] detection probe solution.

Moreover, we carried out interference tests for CRP protein when it coexists with different species, such as Zn^2+^, Ca^2+^, Mg^2+^, urea, and BSA as generally presented saliva composition. Our results indicated that these species had minor interference to CRP detection in a stimulated saliva condition as the control solution (Figure 3c), which indicates that our method based on the formed [ZGO0.5M-Ab-Q] conjugate FRET immunoassay had good selectivity due to the affinity of the CRP antibody and CRP protein. Hence, forming the [ZGO0.5M-Ab-Q] conjugate FRET immunoassay with our ZGO:0.5%Mn^2+^ NPs can be a potential method for salivary CRP detection.

To reach the detection limit of CRP protein for the normal salivary test range (∼3 ng/mL), we carried out multiple measurements for a dilute detection probe solution with a fixed CRP concentration of 2.5 ng/mL. After adding CRP protein, the conjugation solution showed a clear average emission enhancement from the formed [ZGO0.5M-Ab-Q] conjugate FRET immunoassay (Figure 3d). However, at a CRP concentration of 2.5 ng/mL, we observed variation of PL emission recovery using our [ZGO0.5M-Ab-Q] conjugate FRET immunoassay (Figure S20), which indicates that a CRP concentration of 2.5 ng/mL is the detection limit of our [ZGO0.5M-Ab-Q] conjugate FRET immunoassay, and multiple tests are needed for lower CRP concentrations to get accurate results.

To sum, we have successfully demonstrated that our ZGO0.5M NPs with [ZGO0.5M-Ab-Q] conjugate FRET immunoassay can be used as a potential probe for detecting CRP protein in saliva, for both healthy individuals who have no inflammation with low CRP concentrations less than 3 ng/mL and those who have inflammation with CRP level over 10 ng/mL for the moderate elevation. To achieve better performance in the future, more efforts will be made to improve the assay sensitivity and detection range based on the enhancement of PL intensity of ZGOM, concentration optimization of detection conjugation, and orientation of coated Ab.

CRP detection methods vary in sensitivity, specificity, detection limits, time requirements, and associated costs. Traditional CRP assays detect concentrations ranging from 10 to 1000 mg/L,^23^ making them suitable for identifying acute inflammation but less effective for low-level CRP detection necessary in chronic inflammation or cardiovascular risk assessments. High-sensitivity CRP assays measure levels as low as 0.5 mg/L,^24^ facilitating cardiovascular risk assessment; however, these assays are more costly and time consuming. ELISA offer high specificity and sensitivity for precise quantification,^25^ with detection limits as low as 1 μg/L and a linearity range up to 10 mg/L. Despite the accuracy, ELISA requires specialized equipment and longer processing times, which may limit rapid applicability. Immunoturbidimetric assays, utilized in automated analyzers, provide rapid quantification with detection limits around 2 mg/L.^26^ While these assays offer quick results, their sensitivity may not be sufficient for detecting low CRP levels associated with chronic conditions. Advancements in CRP detection include electrochemical immunosensors,^27^ which employ functionalized nanomaterials to achieve detection limits as low as 0.1 nM, offering high sensitivity and potential for point-of-care testing. However, these sensors involve complex fabrication processes and may require careful handling. Quantum dot-based quantitative fluorescence point-of-care testing assays enable rapid CRP quantification within 15 min, with detection limits as low as 0.25 mg/L.^28^ These assays balance speed and sensitivity but face stability issues and require specialized readers. Latex agglutination tests are simple and rapid, providing semiquantitative results without specialized equipment;^29^ however, they have lower sensitivity and specificity, making them less suitable for precise measurements. Immunodiffusion techniques are cost effective and simple but are time consuming and offer less precise quantification,^29^ limiting their utility in urgent clinical settings. Hence, the FRET-based immunoassay built from our luminescent ZGO0.5M NPs as reported above is advantageous for salivary CRP detection compared with these methods.

## Conclusion

To conclude, we successfully synthesized Zn_2_GeO_4_:0.5%Mn^2+^ NPs by a combined coprecipitation and MSS procedure. We also achieved high luminescence QY for these NPs after optimizing the synthesis procedure, i.e., coprecipitating precursors at pH 10 by NH_4_OH, MSS temperature at 900 °C, precursor to salt ratio at 1:10:10 (precursor:NaCl:KCl), and a Mn^2+^ doping level of 0.5%. The ZGO:0.5%Mn^2+^ NPs synthesized under the optimized conditions demonstrated sufficient photoluminescence performance upon UV exposure. We obtained a maximum quantum yield of 34.3% from these optimized ZGO:0.5%Mn^2+^ NPs with green emission upon UV excitation. Moreover, we demonstrated the potential bioapplications of the synthesized ZGO:Mn^2+^ NPs by carrying out a FRET-based immunoassay for salivary CRP detection. We confirmed that they are suitable for salivary CRP detection in the range of 5–20 ng/mL for moderate elevation along with the qualitative test of 2.5 ng/mL of CRP for health (normal) level detection.

## Supplementary Material



## Notes

Experimental details on the synthesis and characterization of ZGOM NPs and FRET-based immunoassay built from the ZGOM NPs for salivary CRP detection (PDF)
