# Supplementary material for: Luminescent Zn2GeO4:Mn2+ Nanoparticles with High Quantum Yield for Salivary Protein Detection
Source: ACS Appl Nano Mater. 2025 Aug 10;8(33):16260–6. doi: 10.1021/acsanm.5c02725 (PMC12379140; doi:10.1021/acsanm.5c02725)
Supplement: Supplementary file 1 [file an5c02725_si_001.pdf]

Supporting Information

Luminescent $Zn_2GeO_4:Mn^{2+}$ nanoparticles with high quantum yield for salivary protein detection

Yuchen Zhang[a] and Yuanbing Mao[a*]

[a]Department of Chemistry, Illinois Institute of Technology, Chicago, IL 60616, USA

*To whom correspondence should be addressed: Email: ymao17@iit.edu, phone: +1-312-567-3815

S1 Synthesis and Characterization of ZGOM NPs by MSS Process

S1.1 Materials. The precursors used to synthesize the ZGOM NPs were zinc nitrate hexahydrate ($Zn(NO_3)_2·6H_2O$, Alfa Aesar, 99%), germanium(IV) dioxide (GeO_2, Acros Organics, 99.999%), manganese(II) chloride tetrahydrate ($MnCl_2·4H_2O$, Sigma-Aldrich), sodium chloride (NaCl, Sigma-Aldrich, ≥99%), and potassium chloride (KCl, Sigma-Aldrich, ≥99%). Ammonium hydroxide (NH_4OH, J.T. Baker, 28.0-30.0%) was used to adjust the pH of the reaction media. Milli-Q water was used as the solvent for precipitating the precursors for MSS synthesis and dissolving the used salts.

S1.2 Synthesis of ZGOM NPs by MSS. ZGOM NPs were synthesized according to the following procedure: First, GeO_2 (1 mM), $Zn(NO_3)_2·6H_2O$ (2 mM), $MnCl_2·4H_2O$ (0.5% mM), NaCl (10 mM) and KCl (10 mM) were dissolved in 10 mL of Milli-Q water, stirring vigorously for 60 min. Next, the pH of the mixture was adjusted to 10.0 with NH_4OH(aq). The resulting solution was vigorously stirring for another 2 h before solvent evaporation process. After stirring, the resulting solution was put into an oven at 80 °C (or on a heating plate at 100 °C) and dried overnight. The resulting dried powder of $(Zn,Mn)_xGeO_y(OH)_z·nH_2O$ was mixed with NaCl-KCl (50%-50% in molar ratio) and finely ground in a mortal, and then transferred to an alumina crucible with a lid and heated up to 900 °C for 2h in muffle furnace with heating and cooling rate at 10 °C/min. After cooling to room temperature, the resulting mixture was added into copious amount of Milli-Q water to dissolve the salt medium. The resulting suspension was washed three times with distilled water and ethanol in a centrifuge (Sorvall ST 8 Small Benchtop Centrifuge) at 8500 rpm for 10 min.

Finally, the precipitate powder was re-dispersed in Milli-Q water or dried at 60 °C for further analyses.

S1.2.1 Effect of Mn^{2+} doping concentration

We first optimized Mn^{2+} doping concentrations into the ZGO:x%Mn^{2+} NPs based on the optimized MSS process, i.e., 900 °C for 2h with precursors coprecipitated at pH 10 with a ratio of precursor to salt at 1:10:10 (precursors : NaCl : KCl). The XRD patterns from the synthesized ZGO:x%Mn^{2+} samples with x = 0.1, 0.2, 0.5, 1.0 and 2.0 (Figure S1a) showed that no impurity phases, including ZnO phase, were presented in the ZGO:0.5%Mn^{2+} sample. However, ZnO impurity phase presented at other ZGO:x%Mn^{2+} samples, such as ZGO:0.2%Mn^{2+}, which might be attributed to the evaporation of germanium precursor during the synthesis process.

PL spectra of the synthesized ZGO:x%Mn^{2+} samples (Figure S1b) demonstrated that the ZGO:0.5%Mn^{2+} sample showed the highest PL emission at 535 nm from Mn^{2+} luminescence with excitation at 254 nm, which also matched with previous studies.[1] These results confirmed that concentration quenching happened at Mn^{2+} doping level higher than 1.0% while PL intensity of Mn^{2+} enhanced as its doping concentration increased up to 0.5% among the synthesized ZGO:x%Mn^{2+} samples. Hence, based on both XRD and PL data, we focused on optimizing the MSS synthesis conditions for the ZGO:0.5%Mn^{2+} NPs below.

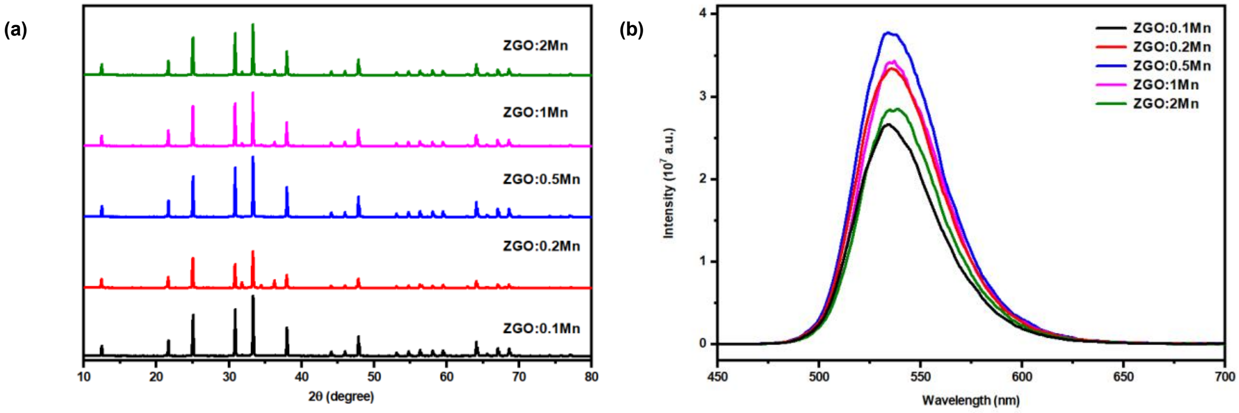

Figure S1. The ZGO:x%Mn^{2+} NPs doped with different Mn^{2+} concentrations (x = 0.1, 0.2, 0.5, 1.0 and 2.0) synthesized by the MSS process of the precursors coprecipitated at pH 10 with a ratio of precursor to NaCl and KCl salts at 1:10:10 at 900 °C for 2h: (a) XRD patterns and (b) PL spectra (λ_{ex} = 254 nm).

S1.2.2 Effect of MSS reaction temperature

Second, we determined the optimized temperature of MSS reaction in the range from 700-1000 °C for the ZGO0.5M NPs from the precursors coprecipitated at pH 10 with a molar ratio of the precursor to salt at 1:10:10 (precursors: NaCl: KCl). Based on the XRD patterns from synthesized samples (Figure S2a), pure ZGO0.5M NPs formed when the MSS temperature was above 900 °C while peaks from ZnO impurity coexist with those from the ZGO0.5M NPs when the MSS temperature was lower than 800 °C meaning unfinished MSS reaction under this condition. The calculated crystallite size using the Scherrer equation from the ZGO0.5M NPs synthesized at 900 °C from the width at half maximum of the peak at 2θ of 33.4° was ~72 nm. Large size particles could be formed by agglomeration during heat treatment and showed particle size larger than calculated (Figure 1c and Figure S7).

PL spectra of these ZGO0.5M NPs synthesized at different MSS temperatures (Figure S2b) indicate that the samples synthesized at 800 and 900 °C shows the highest PL emission intensity at the peak position of 535 nm from the energy transition of $^4T_1$ to $^6A_1$ of Mn^{2+} luminescence. The high intensity of 800 °C sample may be attributable to the ZnO impurity coated outside of nanoparticle which enhanced the luminescence performance.[2] Considering the purity of the synthesized ZGO0.5M NPs from the XRD results, we determined that 900 °C would be the most suitable MSS temperature for synthesizing ZGO0.5M NPs in the rest of this work.

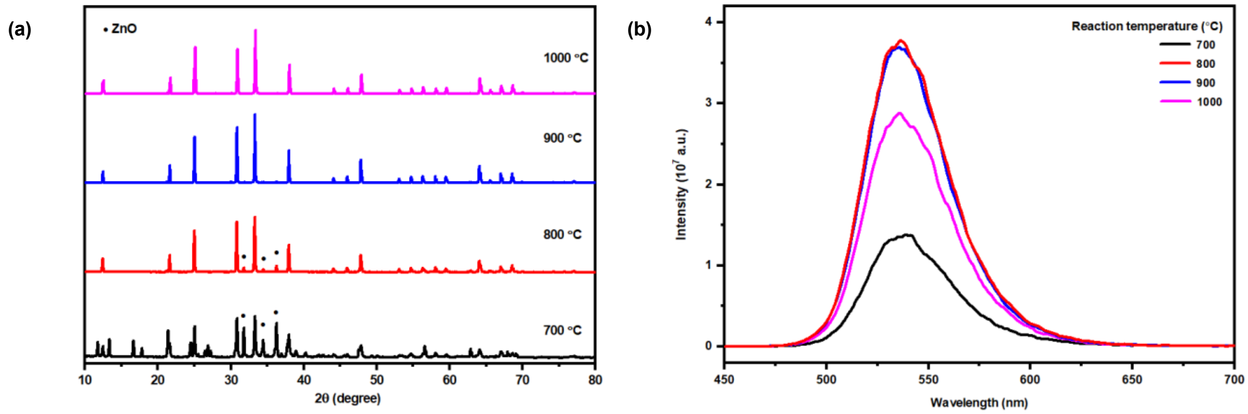

Figure S2. The ZGO0.5M NPs synthesized at different MSS temperatures of 700, 800, 900, and 1000 °C for 2h from the precursors coprecipitated at pH 10 with a ratio of precursor to NaCl and KCl salts at 1:10:10. (a) XRD patterns and (b) PL spectra (λ_{ex} = 254 nm).

S1.2.3 Effect of MSS reaction time

Reaction time test was also performed at 900 °C as shown in Figure S3, ZGO0.5M heating time at 2h achieved pure XRD structure and good PL, which indicated that 2h heating condition is suitable for sample synthesized at 900 °C.

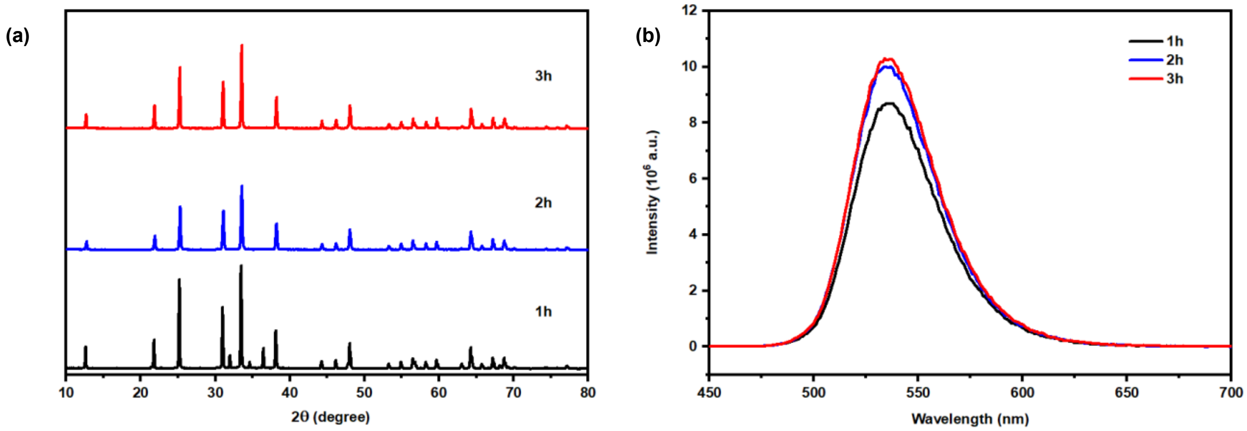

Figure S3. The ZGO0.5M NPs synthesized at different MSS durations of 1, 2 and 3h at 900 °C for 2h from the precursors coprecipitated at pH 10 with a ratio of precursor to NaCl and KCl salts at 1:10:10. (a) XRD patterns and (b) PL spectra (λ_{ex} = 254 nm).

S1.2.4 Effect of coprecipitation pH value

After optimizing the MSS temperature and time, we also studied the influence of coprecipitation pH value on the luminescence properties of the synthesized ZGO0.5M samples. Figure S4a showed the XRD results of the samples synthesized by varying the pH value of the coprecipitation solution. The ZGO0.5M samples synthesized below pH = 10 presented some impurities such as ZnO which might due to the different precipitation intermediates, such as $ZnOH^+$ and $Zn(OH)_2$(aq). At pH at 10, pure ZGO0.5M NPs were formed without ZnO impurity. The ZGO0.5M NPs synthesized from the precursor coprecipitated at pH 10 gave the highest PL intensity (Figure S4b), which might be due to the soluble Ge-species formation at higher pH condition, i.e. from GeO_2 at pH < 9 to H_3GeO^{4-} at pH > 9.[3] Hence, we used the highest achievable solution pH value of 10 using NH4OH aqueous solution as precipitant for coprecipitating precursors, which was employed for the synthesis of the ZGO0.5M NPs in the rest of this work.

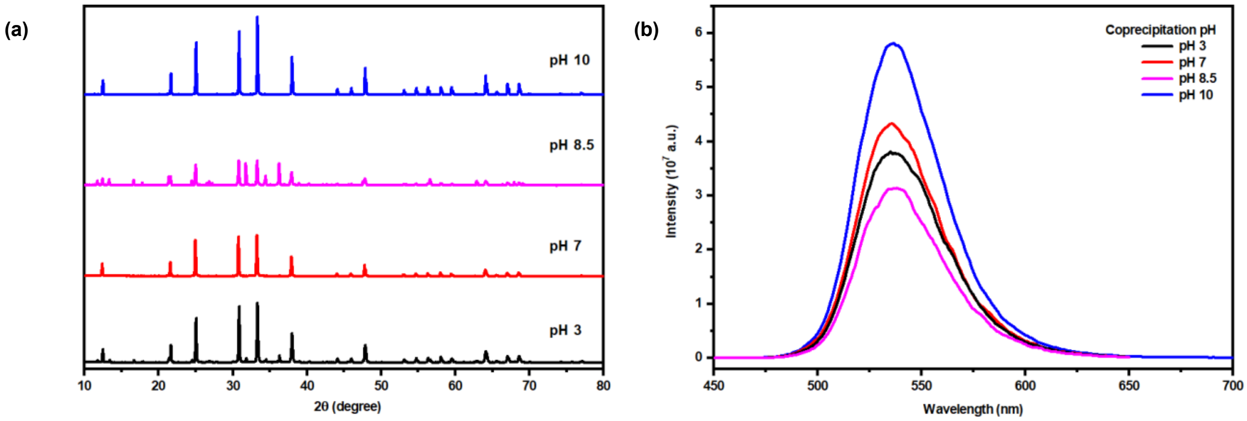

Figure S4. The ZGO0.5M NPs synthesized from the precursors coprecipitated at different pH values of 3, 7, 8.5 and 10 by the MSS process with a ratio of precursor to NaCl and KCl salts at 1:10:10 at 900 °C for 2h: (a) XRD patterns and (b) PL spectra (λ_{ex} = 254 nm).

S1.2.5 Effect of precursor to salt molar ratio

Afterwards, we further investigated the effect of the precursor to salt ratio during the MSS process of the ZGO0.5M NPs on their luminescence properties. Despite the pure crystal structure formed at different precursor to salt ratios in Figure S5a, products from different precursor to salt ratio gave different crystallinity and phase preferences. Products obtained from the precursor to salt ratio of 1:1:1 showed (220) planes with majority while those from other ratios showed the general intensity distributions compared to the JCPDS No-110687 of ZGO. The ZGO0.5M NPs synthesized with the precursor to salt ratio at 1:10:10 had the highest PL intensity of Mn^{2+} emission (Figure S5b). Too high ratio of precursor to salt, e.g., the precursor to salt mixture of NaCl and KCl at 1:1:1 may prevent sufficient solvation of the coprecipitated precursor during the salt melting process. The coprecipitated precursor was not homogeneously solvated in the molten salt medium. On the other hand, the precursor to salt ratio at 1:30:30 gave a dilute and non-homogenous synthesis medium for Mn^{2+} dopant to be sufficiently substituting Zn^{2+} sites nor uniformly distributed within the ZGO host during the MSS process, which contribute to reduced PL intensity.[2] Moreover, precursor to salt ratios of 1:7.5:7.5 and 1:15:15 samples were tested. Minor or no difference in terms of XRD and PL data were observed from these two samples compared to the ZGO0.5M NPs synthesized with the precursor to salt ratio at 1:10:10 under the same conditions. Thereafter, a precursor to salt ratio at 1:10:10 was used as optimized value for the MSS of ZGO0.5M NPs in this study.

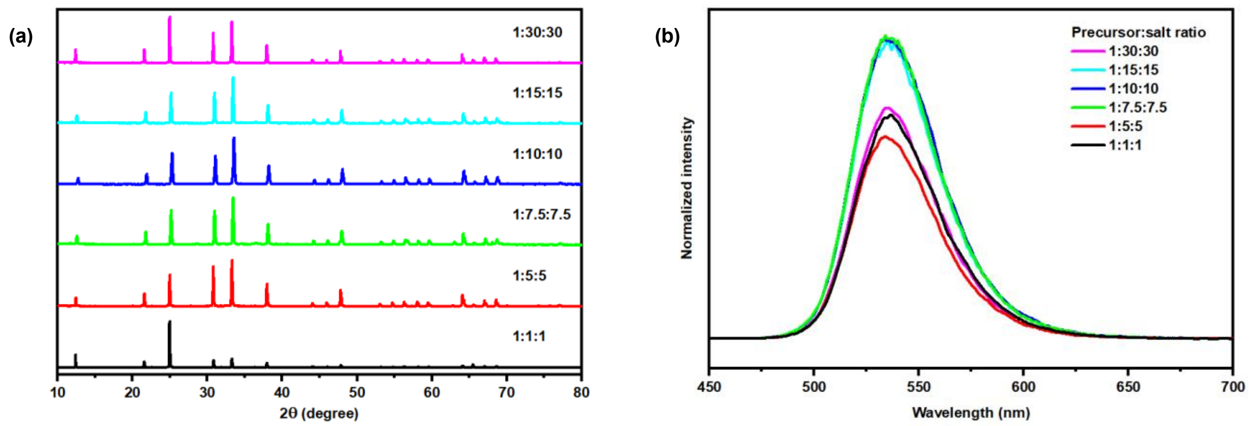

Figure S5. The ZGO:0.5Mn^{2+} NPs synthesized with different ratios of the coprecipitated precursor to salt mixture of NaCl and KCl at 1:1:1, 1:5:5, 1:7.5:7.5, 1:10:10, 1:15:15 and 1:30:30 by the MSS process of the precursors coprecipitated at pH 10 at 900 °C for 2h: (a) XRD patterns and (b) PL spectra (λ_{ex} = 254 nm).

S1.3 Characterization of MSS-synthesized ZGOM NPs. The crystallographic structure was identified by X-ray powder diffraction (XRD) patterns recorded in a Bruker D2 phaser with the step size as 0.02° (2θ) and the step duration of 0.5 s. Scanning electron microscope (SEM) images and energy-dispersive X-ray spectra (EDS) were obtained by JEOL IT800HL SEM at Argonne National Laboratory. Transmission electron microscope (TEM) image and Selected Area Electron Diffraction (SAED) were measured by JEM-2100F TEM at Argonne National Laboratory. Raman spectra were used Renishaw inVia Reflex Raman Microscope at Argonne National Laboratory. Dynamic light scattering (DLS) measurements were performed in aqueous dispersion of the NPs at a concentration of ~0.1 mg·mL^{-1}) using a Malvern Zetasizer Nano-ZS.

PL emission and PL excitation (PLE) spectra for the synthesized ZGOM NPs were carried out in an Edinburgh FLS1000 spectrofluorometer (Edinburgh Instruments) with Xe lamp as excitation source. The excitation and emission wavelengths were 254 and 535 nm, respectively. The luminescence properties of both dried and aqueous suspension samples were measured. Their QY was measured using the same equipment with integration sphere set-up at λ_{ex} of 254 nm and λ_{em} of 535 nm. The absolute PL QY is given by the ratio of the number of photons emitted over the number of photons absorbed, which can be calculated by the excitation and emission area differences between that of the Zn_2GeO_4:0.5%Mn^{2+} NPs and the reference of $BaSO_4$ using an FLS1000 spectrometer software. PersL decay curves were also measured using the same FLS1000

equipment with the detector set at 535 nm, and the samples of our synthesized ZGOM NPs were first charged at 254 nm for 5 min with a UV lamp (UVP UVGL-25 Ultraviolet Lamp). Thermoluminescence (TL) spectra were taken using the same FLS1000 with an externally connected temperature-modulation set-up (THMS600, Linkam) at a sample heating rate of 100 °C/min at temperature range of 300-700 K. Before TL measurements, the sample was preheated to discharge all pre-existing electrons inside of traps. A portable UV lamp was used to charge the sample at 254 nm for 5 min.

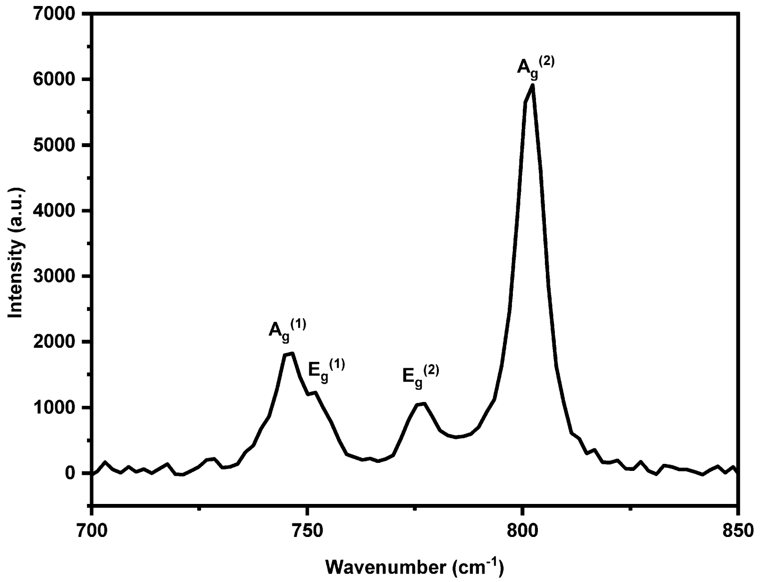

Figure S6. Raman spectrum of the ZGO:0.5Mn^{2+} NPs synthesized at the optimized conditions.

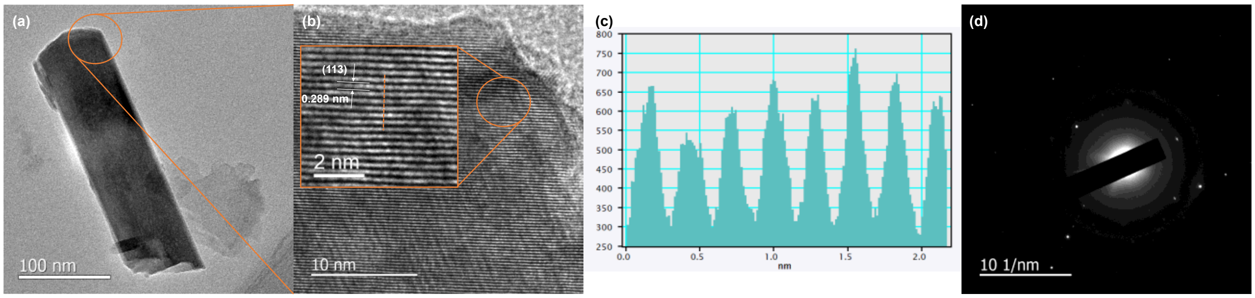

Figure S7. (a) TEM, (b) HRTEM, (c) line profile from the annular bright-field HRTEM image, (d) SAED pattern of the ZGO:0.5Mn^{2+} NPs synthesized at the optimized conditions.

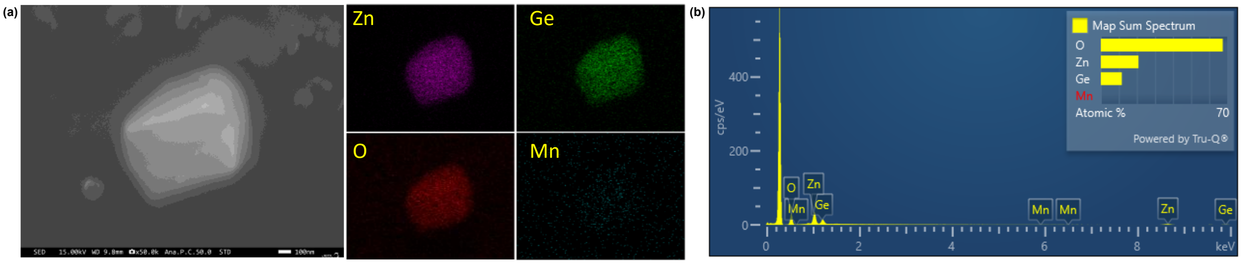

Figure S8. (a) SEM and EDS mapping, (b) EDS spectrum with O, Zn, Ge and Mn elements of the ZGO:0.5Mn^{2+} NPs synthesized at the optimized conditions.

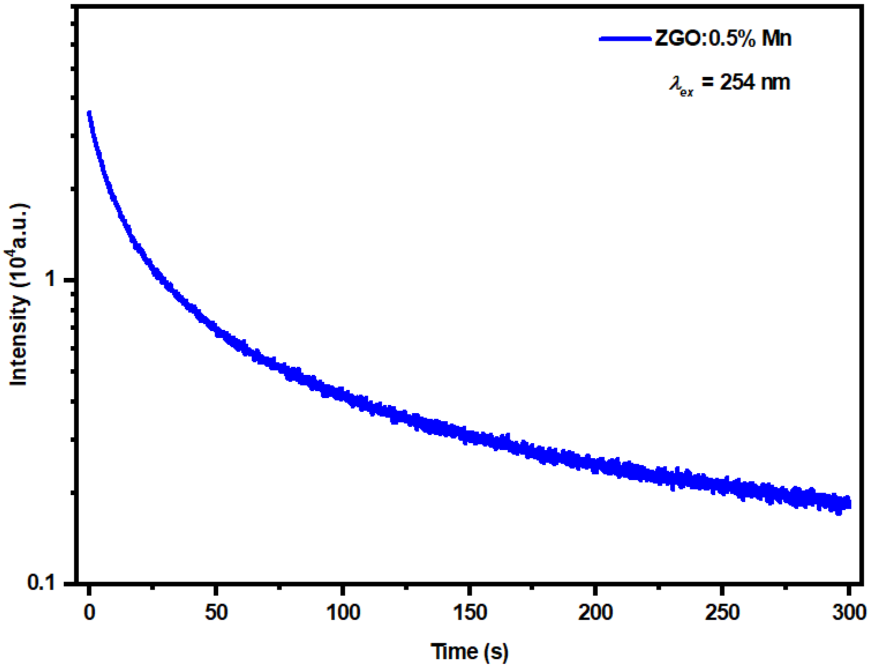

Figure S9. PersL of the ZGO0.5M NPs upon charged at 254 nm for 5 min before the decay test.

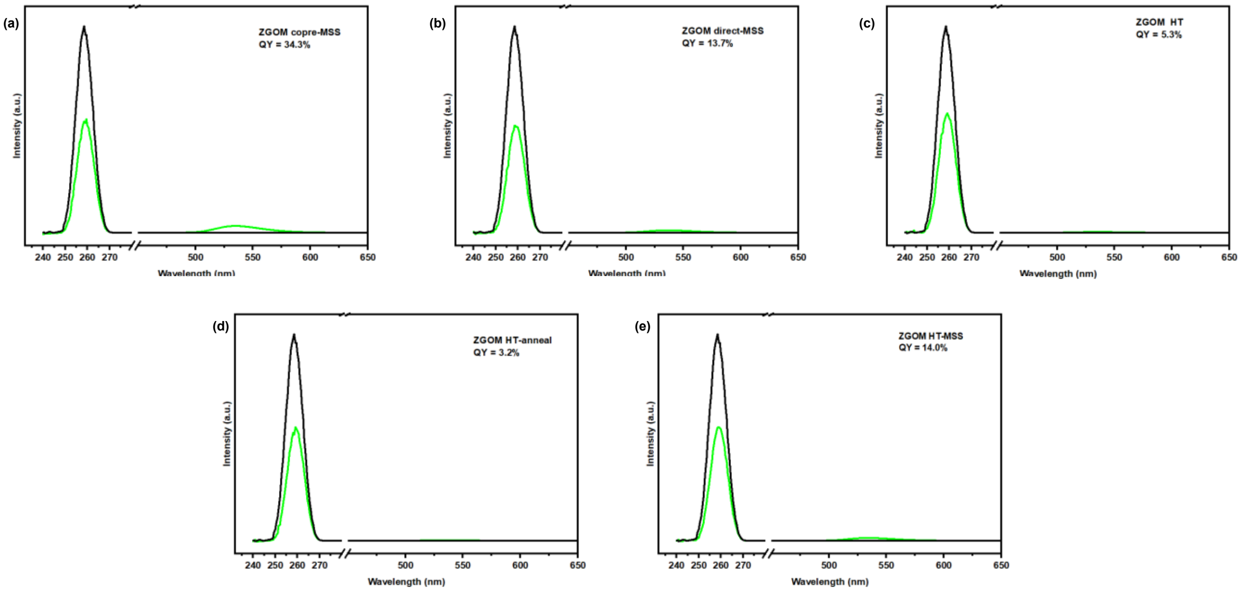

Figure S10. QY values of the ZGO0.5M samples synthesized by different methods: (a) copre-MSS, (b) direct MSS, (c) hydrothermal, (d) HT-anneal, and (e) HT-MSS. λ_{ex} = 258 nm.

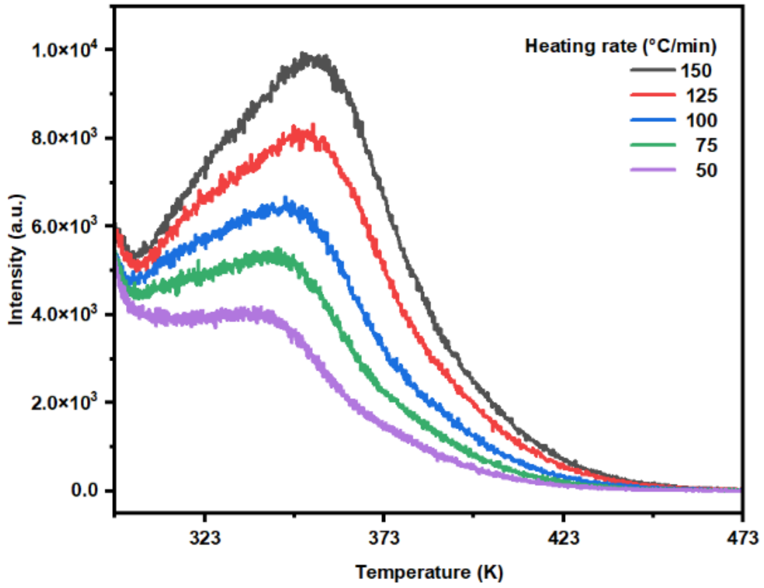

Figure S11. TL spectra of the ZGO0.5M NPs at different heating rates ranging 50-150 °C/min. Formular $E = T_m/500$ was used to calculate the trap depth, where E is the trap depth and T_m is the temperature (K) at maximum peak position. When used TL at the heating rate of 100 °C/min, T_m is 358K, E = 358/500 = 0.72 eV.

S2 Synthesis and Characterization of ZGOM NPs by Other Methods

S2.1 Synthesis of ZGO:0.5%Mn-HT NPs:

ZGO:0.5%Mn-HT NPs were synthesized according to the following procedure based on the literature article with modification:[1] First GeO_2 (1 mM), $Zn(NO_3)_2·6H_2O$ (2 mM) and $MnCl_2·4H_2O$ (0.5% mM) were dissolved in 15 mL of Milli-Q water, stirring vigorously for 60 min. Next, the pH of the mixture was adjusted to 10.0 with NH_4OH. The resulting solution was vigorously stirring for another 1 h. After stirring, the resulting solution was put into autoclave with totally solution volume 17 mL and heat up to 200 °C for 4 h. After cooling to room temperature, the resulting suspension was washed three times with distilled water and ethanol in a centrifuge (Sorvall ST 8 Small Benchtop Centrifuge) at 8500 rpm for 10 min. Finally, the precipitate powders were re-dispersed in Milli-Q water or dried at 60 °C for further analyses.

S2.2 Synthesis of ZGO:0.5%Mn-HT-anneal NPs:

ZGO:0.5%Mn-HT-anneal NPs were synthesized according to the following procedure: The resulting dried powder of ZGO:0.5%Mn-HT described above was ground in a mortal and transferred to an alumina crucible with a lid and heated up to 900 °C for 2h in muffle furnace with heating and cooling rate at 10 °C/min. After cooling to room temperature, the resulting powder was added into copious amount of Milli-Q water. The resulting suspension was washed three times with distilled water and ethanol in a centrifuge (Sorvall ST 8 Small Benchtop Centrifuge) at 8500 rpm for 10 min. Finally, the precipitate powders were dried at 60 °C for further analyses.

S2.3 Synthesis of ZGO:0.5%Mn-HT-MSS NPs:

ZGO:0.5%Mn-HT-MSS NPs were synthesized according to the following procedure: The resulting dried powder of the ZGO:0.5%Mn-HT was mixed with NaCl (10 mM) and KCl (10 mM) in a mortal and ground before transferred to an alumina crucible with a lid and heated up to 900 °C for 2h in muffle furnace with heating and cooling rate at 10 °C/min. After cooling to room temperature, the resulting mixture was added into copious amount of Milli-Q water to dissolve the salt medium. The resulting suspension was washed three times with distilled water and ethanol in a centrifuge (Sorvall ST 8 Small Benchtop Centrifuge) at 8500 rpm for 10 min. Finally, the precipitate powders were dried at 60 °C for further analyses.

S2.4 Synthesis of ZGO:0.5%Mn-MSS NPs:

ZGO:0.5%Mn-MSS NPs were synthesized according to the following procedure: GeO_2 (1 mM), $Zn(NO_3)_2 \cdot 6H_2O$ (2 mM), $MnCl_2 \cdot 4H_2O$ (0.5% mM), NaCl (10 mM) and KCl (10 mM) were finely ground in a mortal and transferred to an alumina crucible with a lid and heated up to 900 °C for 2h in muffle furnace with heating and cooling rate at 10 °C/min. After cooling to room temperature, the resulting mixture was added into copious amount of Milli-Q water to dissolve the salt medium. The resulting suspension was washed three times with distilled water and ethanol in a centrifuge (Sorvall ST 8 Small Benchtop Centrifuge) at 8500 rpm for 10 min. Finally, the precipitate powders were dried at 60 °C for further analyses.

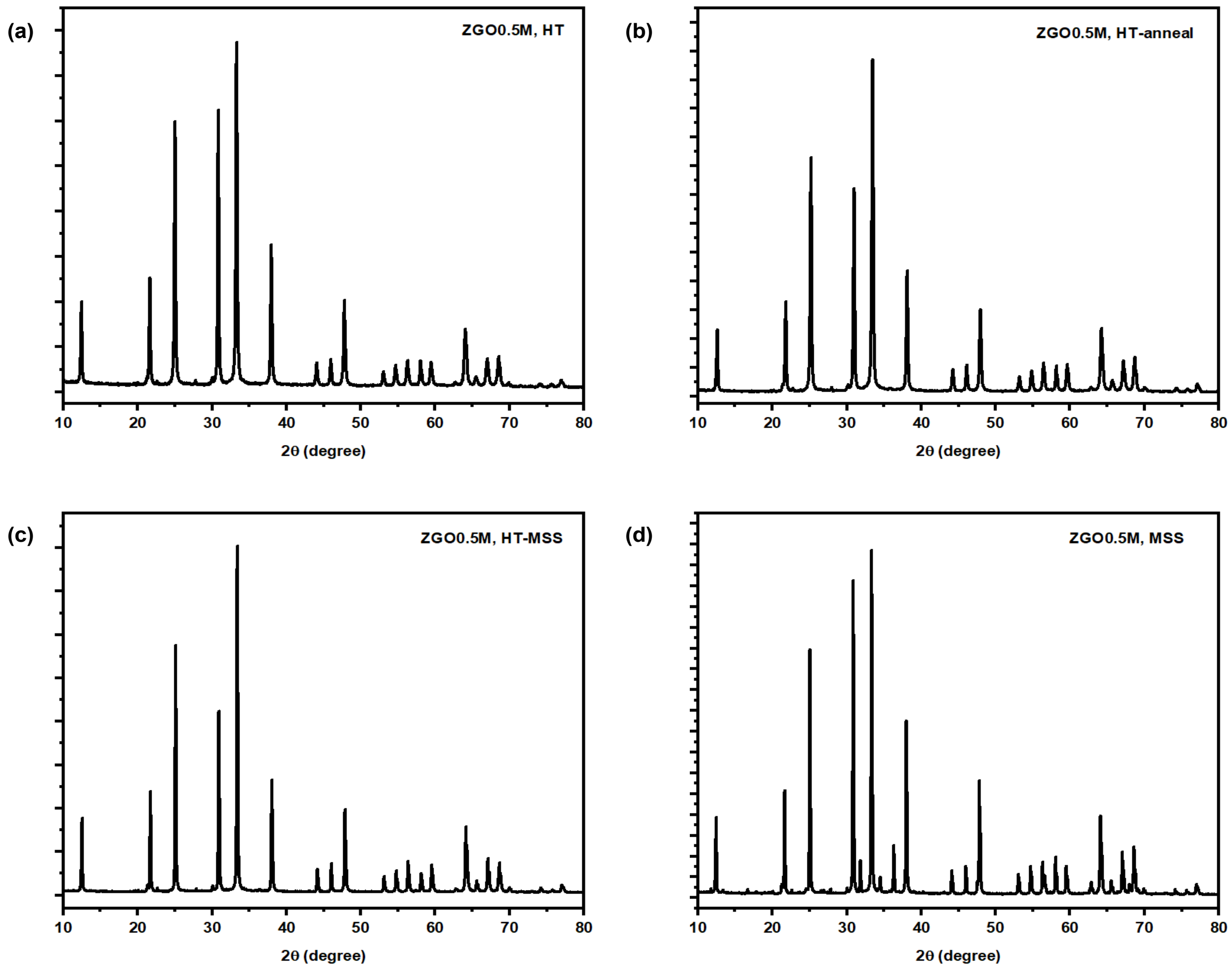

Figure S12. XRD patterns of the Zn_2GeO_4:0.5%Mn samples synthesized using other methods: (a) hydrothermal, (b) hydrothermal-anneal, (c) hydrothermal-MSS and (d) direct MSS methods. Samples used same reaction temperature and time except hydrothermal method.

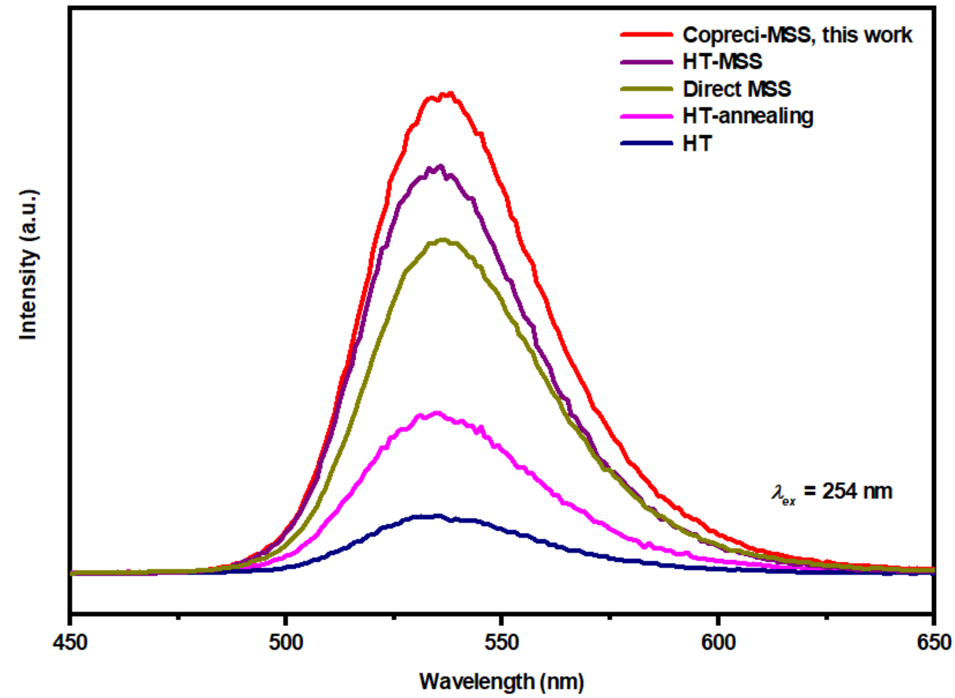

Figure S13. Comparison of PL spectra (λ_{ex} = 254 nm) of the ZGO:0.5%Mn samples synthesized by different methods.

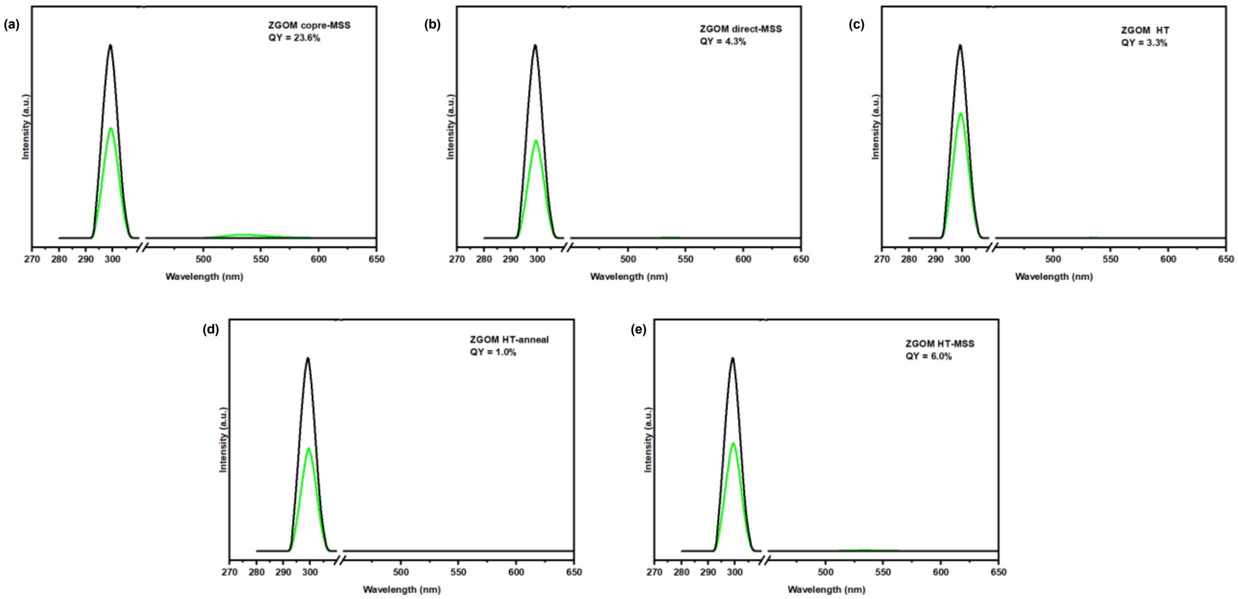

Figure S14. QY of the ZGO0.5M samples synthesized by different methods: (a) copre-MSS, (b) direct MSS, (c) hydrothermal, (d) HT-anneal, and (e) HT-MSS. λ_{ex} = 300 nm.

Table S1. A comparison of the ZGO:Mn NPs synthesized by different methods measured at λ_{ex} = 258 nm and 300 nm

	QY@258nm, %	QY@300nm, %
Copre-MSS, this work	34.3	23.6
Direct MSS	13.7	4.3
HT	5.3	3.3
HT-anneal	3.2	1.0
HT-MSS	14.0	6.0

S3 FRET-Based Immunoassay built from the ZGOM NPs for Salivary CRP Detection

S3.1 Chemicals, Reagents, and Buffer Solutions. Tetraethyl orthosilicate (TEOS, Sigma-Aldrich), (3-Aminopropyl) triethoxysilane (APTES, Sigma-Aldrich) and diglycolic anhydride (DGA, Sigma-Aldrich) were used to surface coating and modify the ZGOM NPs. N-hydroxylsulfoccinimide (NHS, Sigma-Aldrich) and N-Ethyl-N′-(3-dimethylaminopropyl) carbodiimide hydrochloride (EDC, EMD Millipore Corp) were used to couple CRP antibody with the ZGOM NPs. Phosphate buffered saline (PBS, Sigma-Aldrich, P4417-50TAB) was used to generate pH = 7.4 buffer solution. Bovine serum albumin (BSA, Sigma-Aldrich) was used as protection/quench agents. CRP protein (Bio-rad, 1707-2029) and CRP antibody (Mouse anti Human CRP, Bio-rad, MCA6186GA) were used to achieve antigen-antibody interaction.

S3.2 Surface modification of the ZGOM NPs. First, the ZGOM NPs were coated with a functional group $-NH_2$ to achieve better absorption with the antibody-quencher pair due to the surface negative charge of the antibody-quencher pair and surface positive charge of the coated ZGOM NPs-NH_2 using the following protocol: We first dispersed 20 mg ZGOM NPs in 10 mL ethanol, sonicated for 5 min, and then added 5 mL NH_4OH solution (after dilution via 1:1 in v:v with H_2O) and sonicated for another 5 min. After vigorously stirring the suspended solution on a hot plate, we added 100 µL of TEOS and 25 µL of APTES into the solution and heated the solution up to 50 °C overnight to achieve $-NH_2$ coating. Finally, the resulting mixture was centrifuged and washed three times to remove any excess reagent. Once the last supernatant was discarded, the coated ZGOM-NH_2 were resuspended in PBS at pH = 6 for further experiments.

S3.3 Homemade $KAuCl_4$ powder:

Generally speaking, $KAuCl_4$ powder were made by first dissolving gold in aqua regia, followed with the evaporation processing of the solution alone with the unreacted acid to concentrate the gold solution. After concentration, KOH were used to adjust pH to neutral. The neutral solution was transferred to oven for the dry process to get $KAuCl_4$ powder for the further synthesis of the Au NPs as described in the main text.

S3.4 Synthesis of gold NPs:

We first synthesized gold NPs following a previous method with modifications.[4] We dissolved ~10 mg homemade $KAuCl_4$ powder in 100 mL of Milli-Q water at pH ~5, heated the solution up to 90 °C with vigorous stirring. We also dissolved 20 mg trisodium citrate in 1 mL of Milli-Q water and then added the resulted solution into the $KAuCl_4$ solution under 90 °C heating condition. After a 10-min reaction, we cooled the resulting solution down in an ice bath. The cooled solution was centrifuged once to remove the excess reagents and resuspended in Milli-Q water solution and kept at 2-6 °C as the gold NP stock solution.

S3.5 Conjugation of CRP antibody-quencher [Ab-Q] pair. Here the as-synthesized gold NPs were used as the quencher (Q) for the coated ZGOM-NH_2 after binding with CRP antibody to achieve [Ab-Q] pair. The conjugation of the as-prepared gold NPs with the CRP antibody was done by physical absorption with the following steps: We first adjusted the stock solution of the gold NPs to pH 10 by 1 M NaOH solution and then added CRP antibody to the resulting gold NPs solution followed with gentle stirring for 30 min at room temperature. We then added the resulting gold NPs-CRP antibody solution to 5% BSA solution and gently stirred it for another 30 min. Finally, we centrifuged the resulting gold NPs-CRP antibody solution with BSA, washed it three times with PBS buffer (pH = 7.4), resuspended it in PBS buffer solution, and kept it at 2-6 °C for further testing.

S3.6 FRET-based immunoassay preparation. The immunoassay preparation protocol as being schematized in Scheme 1 consists of the following steps:

1. To form the detection probe, add the [Ab-Q] antibody-gold pair into the modified ZGOM-NH_2 in PBS solution and gently shake for 30 min to make the [ZGOMn-Ab-Q] conjugate.
2. Dilute the [ZGOMn-Ab-Q] conjugate solution to suitable concentration with PBS (pH = 7.4) solution.

3. Add 1 mL of the standard CRP test solution into 1 mL of the [ZGOMn-Ab-Q] conjugate solution and gently shake for about 15 min.

4. Use the same process for CRP testing samples with unknow concentration. If it reaches full recovery intensity, then dilution would be needed for the testing samples.

5. Measure the PL intensity of CRP testing samples using fluorescence spectrometer with 1cm*1cm quartz cuvette.

6. For qualitative test, detection probe solution was dilute into ¼ for the testing.

S3.7 Instrumentation. PL and PLE spectra were measured by a FLS 1000 spectrofluorometer with quartz sample cuvette of 1×1 cm with sample volume around 1.2-2 mL.

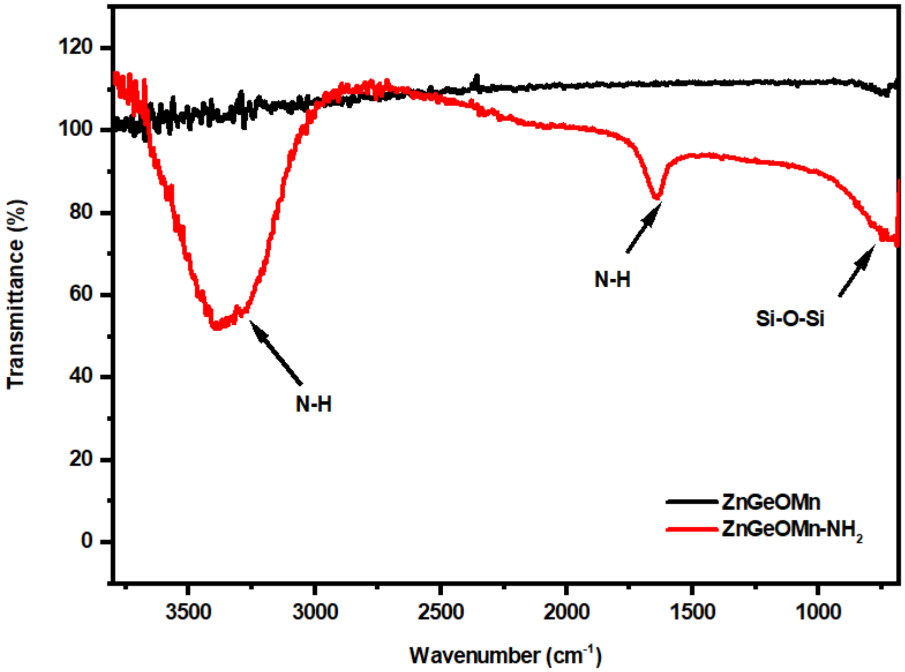

Figure S15. FTIR spectra of the ZGO:0.5%Mn NPs before and after $-NH_2$ coating. Insert arrows indicate the stretching mode of N-H at ~3400 cm^{-1}, bending mode of N-H at ~1600 cm^{-1}, and potential Si-O group at range below 1000 cm^{-1}.

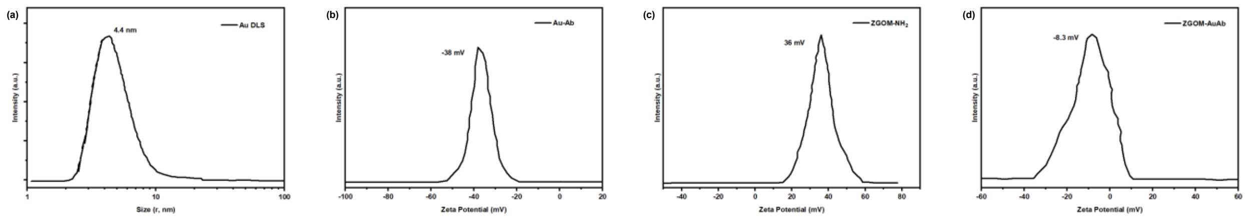

Figure S16. DLS and zeta potential spectra of the Au NPs and ZnGeOMn NPs used volume-based distributions. Au NPs and ZGOM NPS were dispersed in Milli-Q water with optimized concentration. (a) DLS of Au NPs, (b) Zeta potential of the Ab-Au, (c) Zeta potential of the ZGOM-NH_2 NPs, and (d) Zeta potential of the ZGOM-AbAu conjugate.

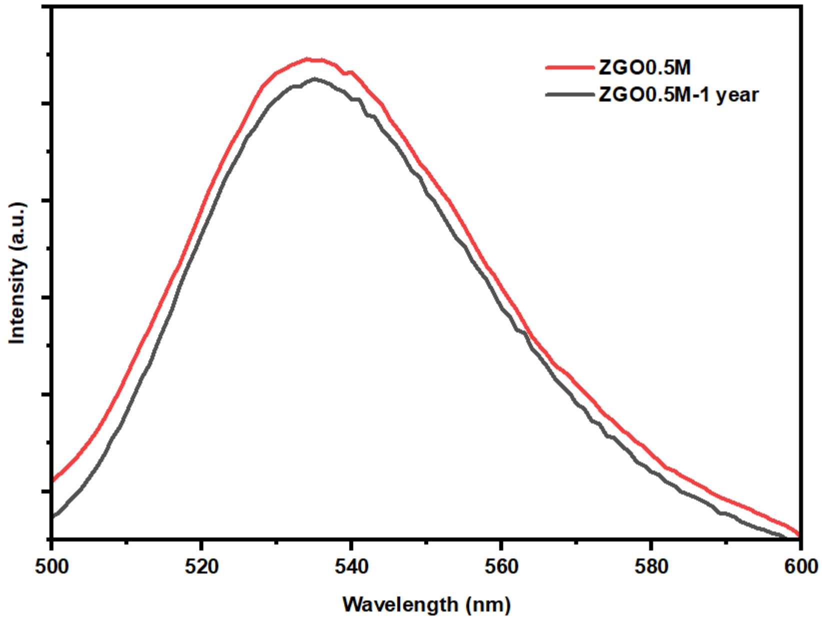

Figure S17. Long time stability test of ZGO0.5M. Storage in 2-6 ℃.

S3.8 Confirmation of strong interaction between Au NPs and CRP-antibody

Upon coating with the CRP-antibody, the surface of the Au NPs was modified to form Au-S bond, which could prevent the Au NPs from agglomeration upon introducing high ionic solutions. Figure S18a showed Au NPs agglomerated after 10% NaCl solution injection. After coating with CRP-antibody, our Au NPs did not change the color even after injecting 10% NaCl solution (Figure S18b). However, when the coating process happened at lower pH environment ($pH < 9$) (Figure S18c), less Ab used (25%) for coating (Figure S18d) and no Ab used for coating (Figure S18e) were showed the color change after 10% NaCl injection, which indicated that the high pH environment and Ab were the requirement for the surface coating of the ZGOM NPs. Figure S18f showed a typical absorption spectrum of the Au NPs with (gray line) and without (red line) agglomeration.

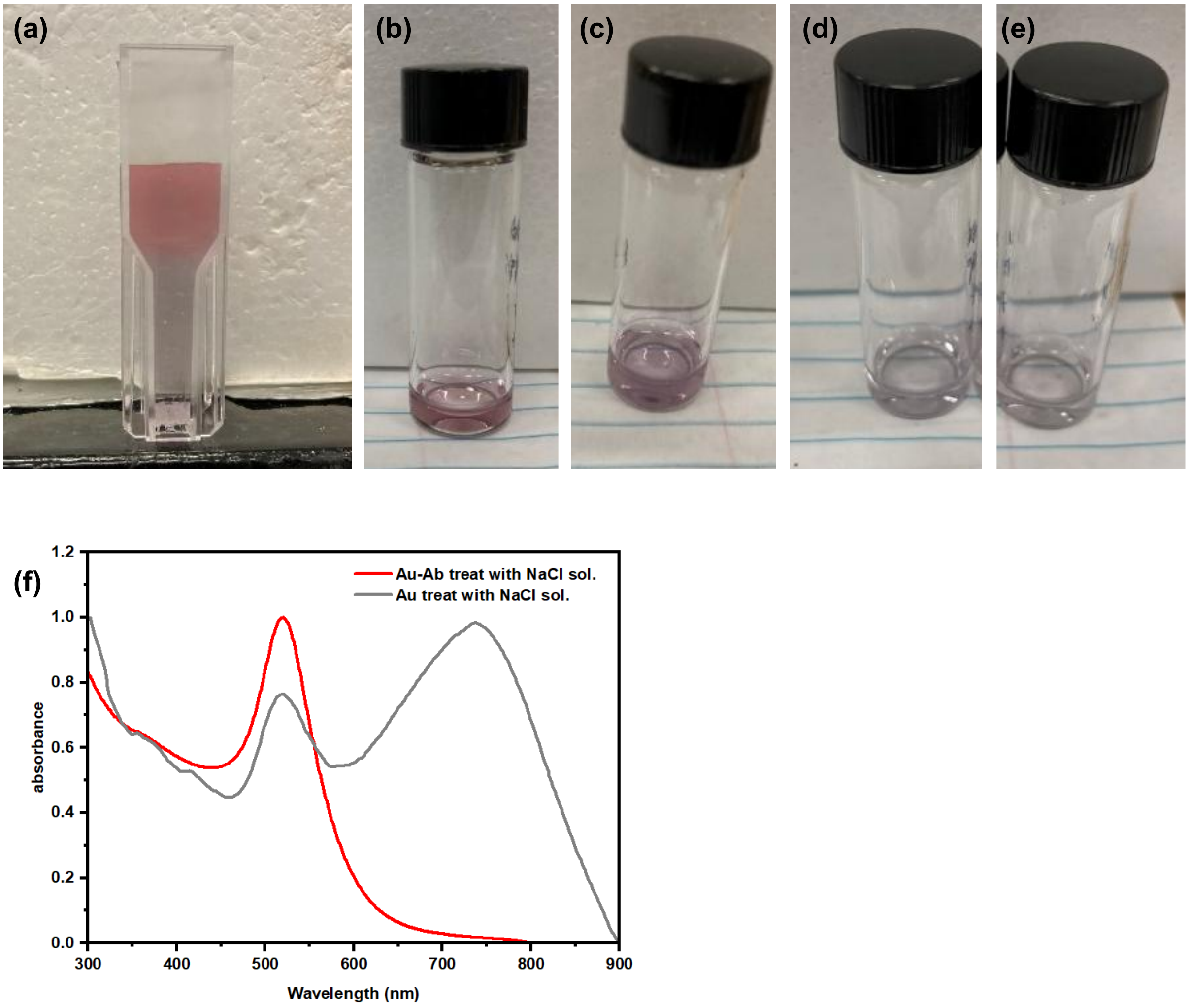

Figure S18. Stability test of the Au NPs coated with CPR antibody by 10% NaCl solution. (a) Color change by mixing 10% (w/w) NaCl aqueous solution with the Au NPs solution, top part is the pure Au NPs solution and bottom part is the Au NPs injected with NaCl solution. (b) The color of the Au NPs coated with Ab solution remained the same after adding 10% (w/w) NaCl aqueous solution. (c) Color changed with coating at low pH of Au NPs and Abs could generate partially coated conjugation, however, the aggregation still happened after adding NaCl solution. Full aggregation of the Au NPs due to (d)the less CRP antibody (25%); and (e) the pure Au NPs without CRP antibody. (f) Absorption spectra of samples from Figure S18b and Figure S18c. The mechanism for the stability test was to measure the absorption change of the Au NPs, which controlled by agglomeration of the Au NPs. Antibody coated Au NPs would prevent agglomeration from the 10% NaCl solution and remain the absorption spectrum.

Table S2. Detailed sample information presented in Figure S18

Figure S18	pH	Coating parameter/mL	
**a**	7	200 µg Au	Top: pure Au NPs<br>Bottom: Au NPs + NaCl
**b**	10	200 µg Au + 2 µg Ab	
**c**	8	200 µg Au + 2 µg Ab	
**d**	10	200 µg Au + 0.5 µg Ab	
**e**	10	200 µg Au	

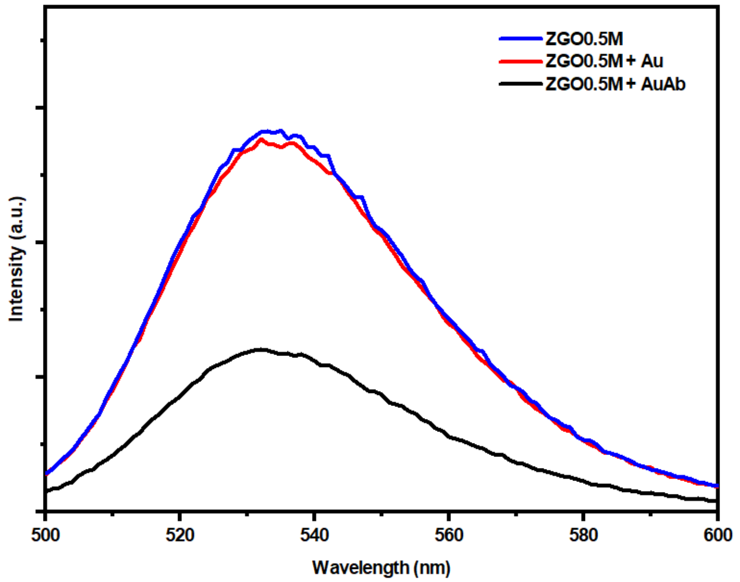

Figure S19. Confirmation of combination between surfaced modified ZGOM and antibody coated gold NPs by comparing the emission spectra of the ZGO0.5M NPs by our conjugation reaction with the AuNPs as reported in this study and by just by direct physical mixing.

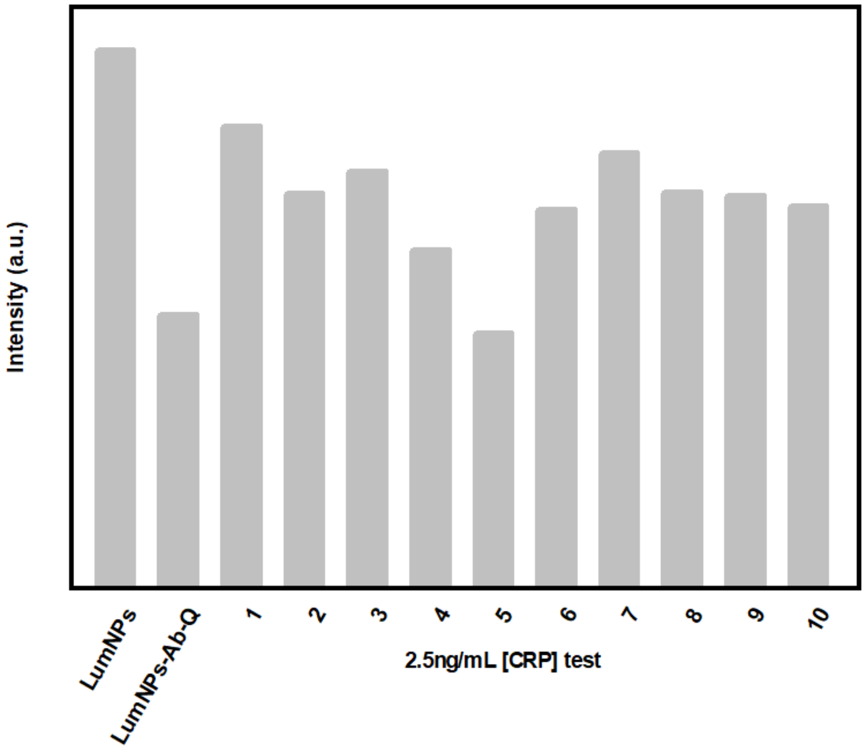

Figure S20. 2.5 ng/mL CRP multiple samples test after dilution by 4 times of the LumNPs-Ab-Q conjugation solution mixed with 10 sets of 2.5 ng/mL of CRP solution

References:

1. Srivastava, B. B.; Gupta, S. K.; Li, Y.; Mao, Y., Bright persistent green emitting water-dispersible Zn_2GeO_4:Mn nanorods. *Dalton Trans.* **2020,** *49* (22), 7328-7340. DOI: 10.1039/d0dt00361a
2. Gupta, S. K.; Mao, Y. B., A review on molten salt synthesis of metal oxide nanomaterials: Status, opportunity, and challenge. *Prog. Mater. Sci.* **2021,** *117,* 100734. DOI: 10.1016/j.pmatsci.2020.100734
3. Nikolaychuk, P. A., The potential - pH diagram for germanium. *Phosphorus Sulfur* **2023,** *198* (9), 705-714. DOI: 10.1080/10426507.2023.2193747
4. Kimling, J.; Maier, M.; Okenve, B.; Kotaidis, V.; Ballot, H.; Plech, A., Turkevich method for gold nanoparticle synthesis revisited. *Journal of Physical Chemistry B* **2006,** *110* (32), 15700-15707. DOI: 10.1021/jp061667w
